# Temporally segregated subpopulations of CDT and TcdA producer cells of *Clostridioides difficile*

**DOI:** 10.1128/msphere.00186-25

**Published:** 2025-09-03

**Authors:** Sara Ramalhete, Isabel Roseiro, Carolina P. Cassona, Carolina Alves Feliciano, Mónica Serrano, Adriano O. Henriques

**Affiliations:** 1 Instituto de Tecnologia Química e Biológica António Xavier, Oeiras, Portugal; The University of Iowa, Iowa City, Iowa, USA

**Keywords:** *Clostridioides difficile*, binary toxin (CDT), TcdA and TcdB, single-cell analysis

## Abstract

Most strains of *Clostridioides difficile* produce two toxins, TcdA and TcdB, which are mainly responsible for the disease symptoms. TcdA and TcdB are coded for by genes in the pathogenicity locus (PaLoc). Some epidemic strains, however, such as R20291, of ribotype 027, additionally produce a binary toxin, CDT, coded for by genes in the CDT locus (CdtLoc). We analyzed the relationship between transcription of the TcdA and CDT encoding genes in strain R20291 using single-cell analysis with orthogonal fluorescence reporters. We found that most of the cells express *cdtA,* whereas a much smaller fraction expresses *tcdA,* and most of those also express *cdtA*. Expression of *cdtA* begins during exponential growth and persists during the stationary phase of growth, while the main period of *tcdA* transcription occurs as cells enter the stationary phase. This translates into early synthesis and release of CDT from the cells, whereas production of TcdA is detected mainly from the onset of the stationary phase. Both the PaLoc and CdtLoc also code for regulatory proteins, TcdR, a sigma factor, and CdtR, a response regulator of the LytR family, required for expression of the toxin-encoding genes, respectively. While CdtR contributes to the expression of *tcdA*, as shown before, we found reduced expression of *cdtA* in the absence of TcdR. Hence, TcdR and CdtR cross-regulate the PaLoc and the CdtLoc. Finally, and unlike the case for TcdA, we found no evidence for the association of CDT with mature spores.

**IMPORTANCE:**

The enteropathogen *Clostridioides difficile* causes a spectrum of intestinal diseases, ranging from mild diarrhea to severe conditions such as intestinal inflammation, perforation, and sepsis, that may lead to death, primarily through the production of two cytotoxins, TcdA and TcdB. Certain strains, however, such as those of ribotypes 027 and 078, additionally produce a binary toxin, CDT. Here, we employed single-cell analysis to investigate toxin gene expression in epidemic strain R20297 (RT027), commonly associated with severe infections. We found that CDT is synthesized early during growth, while TcdA is produced at the onset of the stationary phase, and that the two populations partially overlap. We also identify cross-regulation between two key regulatory proteins, TcdR and CdtR, which control TcdA/TcdB and CDT production. These insights into the mechanisms of toxin production at the population level may contribute to the development of targeted therapies for managing *C. difficile* infections and the resulting complications.

## INTRODUCTION

*Clostridioides difficile* is a Gram-positive, spore-forming anaerobic bacterium that may asymptomatically colonize the gastrointestinal tract or trigger an infection that is usually preceded by gut dysbiosis. Infection by *C. difficile* (CDI) is characterized by elevated yellow-white plaques that coalesce to form pseudomembranes on the colonic mucosa (pseudomembranous colitis), and it may end up causing a range of diseases from severe diarrhea and toxic megacolon to bowel perforation, sepsis, septic shock, and death (reviewed by references 1, 2).

**Fig 1:**
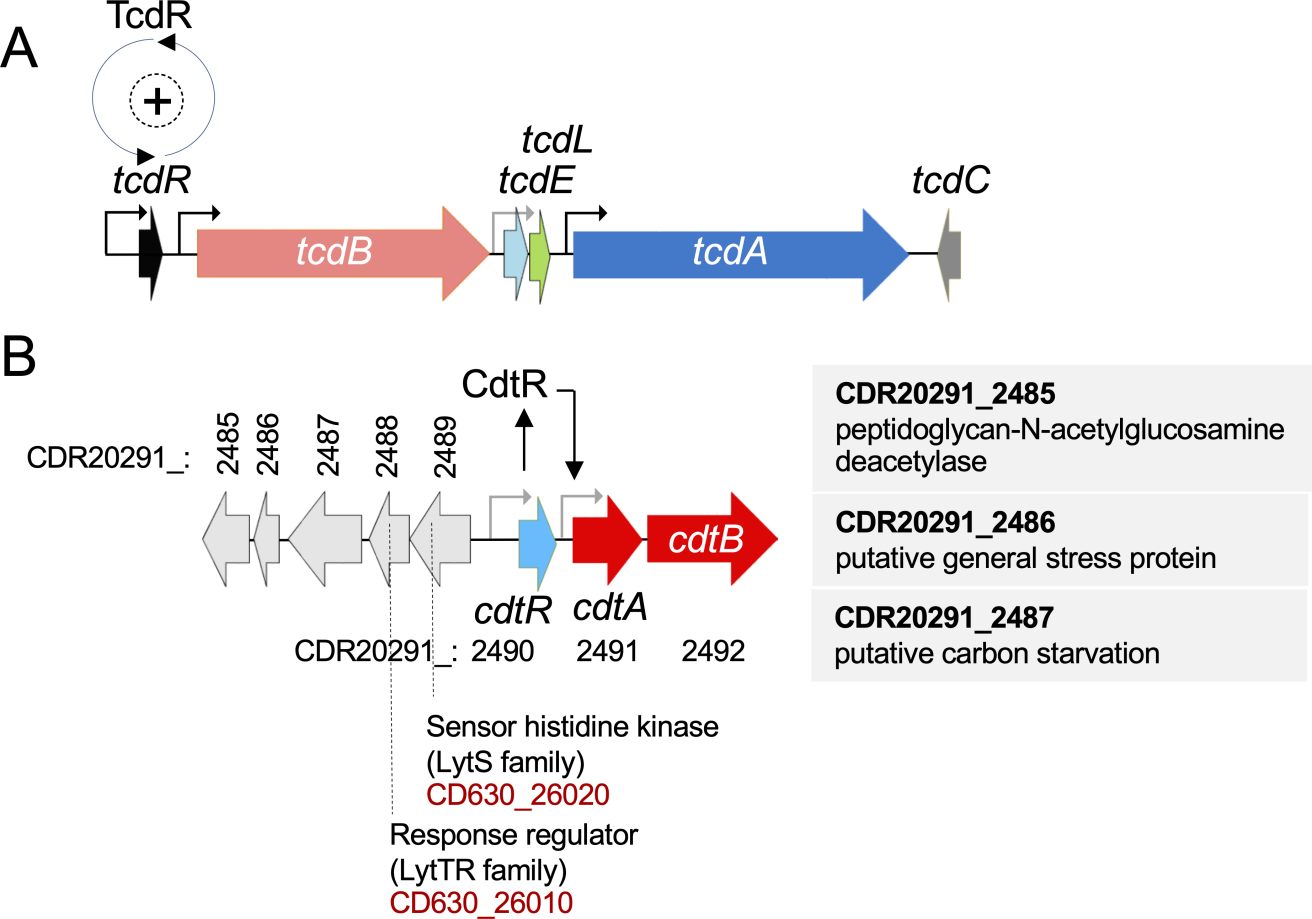
Schematic representation of the PaLoc and the CdtLoc. (**A**) Schematic representation of the pathogenicity locus (PaLoc). TcdR recognizes promoters upstream of *tcdR* (and is thus auto-regulatory), *tcdB,* and *tcdA* (black arrows); a putative TcdR promoter exists upstream of *tcdE* (gray arrow) (3, 4). (**B**) Schematic representation of the binary toxin locus (CdtLoc) of strain R20291. CdtR regulates *cdtA* expression through a direct or indirect pathway (23). The gray arrows represent putative promoters. The CDR20291_2489 gene, upstream of and divergently oriented relative to *cdtR*, codes for a putative LytR family sensor histidine kinase and is followed by a gene coding for a putative transcription factor of the LytR family, CDR20291_2488; these genes are also found in the genome of strain 630Δ*erm*. The codes for all the genes in the region are given (see also the sequence alignment in Fig. S1).

CDI symptoms were shown to result mainly from the activity of two large clostridial glucosylating toxins, TcdA and TcdB, responsible for the disease (1, 2). These toxins are coded for by genes located in a pathogenicity locus (PaLoc), which carries four additional genes, *tcdR*, *tcdE*, *tcdL,* and *tcdC* (Fig. 1A). TcdR is an RNA polymerase sigma factor that serves as the main positive regulator of expression of the PaLoc; *tcdR* is under positive auto-regulation (3, 4). The auto-regulatory loop results in bimodal expression of the toxin-encoding genes and is mainly primed by the flagellar regulator σ^D^ (5, 6). In addition to TcdR, several other regulatory proteins control expression of the toxin-encoding genes (7, 8). TcdA and TcdB have no recognizable secretion signals, and their transport to the outside of the cells involves the PaLoc gene *tcdE*, which codes for a holin-like protein, and a dedicated peptidoglycan amidase, coded for by a gene outside of the PaLoc, that contributes to toxin secretion without causing cell lysis (9–13). *tcdL* codes for a 43 residues-long fragment resembling a non-catalytic fragment of an endolysin that binds to TcdB and may facilitate its transport by an unknown mechanism (11, 14). The toxin-encoding genes are also expressed in a fraction of the sporulating cells, and the toxins associate with spores; thus, release of the mother cell contents and spores at the end of sporulation represents another route for toxin release (6). Finally, *tcdC* codes for a small single-pass transmembrane protein that inhibits TcdR-dependent transcription *in vitro* but whose role *in vivo* is not yet firmly established and may be strain-dependent (15–22).

In addition to TcdA and TcdB, some *C. difficile* strains, including strains belonging to the epidemic ribotype (RT) 027, such as R20291, and the emergent virulent lineage RT078, produce a third toxin known as the binary toxin (CDT; reviewed by reference 2). The CDT toxin is involved in the adhesion of *C. difficile* cells to the gut epithelium as it induces the formation of microtubule protrusions (24); it also suppresses a protective host eosinophilic response (25, 26) and induces the formation of biofilm-like microcolonies (27). CDT is encoded by two genes, *cdtA* and *cdtB,* part of the CDT locus (CdtLoc), that also includes the *cdtR* gene (28) (Fig. 1B). *cdtR* codes for a response regulator of the AlgR/AgrA/LytR family (29) required for expression of the *cdtAB* operon (23, 30, 31). CdtR also regulates TcdA and TcdB production in RT027 strains (32). Although strain 630 has a functional *cdtR* gene, it carries *cdtAB* pseudogenes, and CdtR does not regulate TcdA/TcdB production (32, 33; Fig. 1); the rationale for the presence of a functional *cdtR* is not known.

The closest structural homolog of CdtR is ComE, which regulates competence in *Streptococcus pneumoniae* (34); the phosphor-acceptor residue conserved in response regulators of the AlgR/AgrA/LytR family is present in CdtR and is important for expression of the *cdtA*/*B* genes in *C. difficile* (30) (Fig. S1). CdtR is an orphan response regulator, and the kinase that presumably phosphorylates it has not been identified. Perhaps significantly, two genes just upstream and divergent from the *cdtR* in both strains R20291 and 630 code for another LytR-type response regulator and a LytS-type histidine kinase; these genes, however, have not been characterized (Fig. 1B; Fig. S1A).

Expression of the CdtLoc has not been studied at the single-cell level. Here, we use orthogonal fluorescence reporters to study *cdtR*, *cdtA,* and *tcdA* expression at the single-cell level in strain R20291, a RT027 strain. We show that *cdtR* and *cdtA* are homogeneously expressed across the population and are detected early and throughout growth. In contrast, and in line with previous studies, *tcdA* is heterogeneously expressed, mainly from the onset of the stationary phase onward. Using dual labeling, we also show that the majority of the *tcdA*-expressing cells also express *cdtA*. As expected, CdtR was found to regulate production of both CDT and TcdA (32). Surprisingly, however, we found that TcdR also has an impact on the expression of the *cdtAB* operon. We propose a timeline for TcdA and CdtA production during the *C. difficile* infection cycle.

## RESULTS

### Expression patterns of *tcdA*, *cdtR,* and *cdtA* in strain R20291: timing, levels, and cellular heterogeneity

Although expression of the PaLoc genes was already studied at the single-cell level in strain 630 and others, including R20291, using different fluorescent reporters; in the three studies, expression of *tcdA* was found to be bimodal, with at least 65% of the population of vegetative cells (the number refers to studies using the SNAP^Cd^ reporter) in TY cultures in the ON state (5, 6, 35). Bimodality rests on the auto-regulatory nature of *tcdR* (5). In strain 630, the flagellar switch is locked in the ON state, leading to the production of σ^D^, which primes the TcdR positive feedback loop, allowing expression of *tcdA* in a significant fraction of the population (5; 83% in this study). In R20291, however, the flagellar switch undergoes phase variation, and the fraction of *tcdA* ON cells is down to about 15% (5). Expression of *tcdR* was only reported using the *SNAP^Cd^* reporter and was detected in only 7% of the vegetative cells under the same culturing conditions (6).

**Fig 2:**
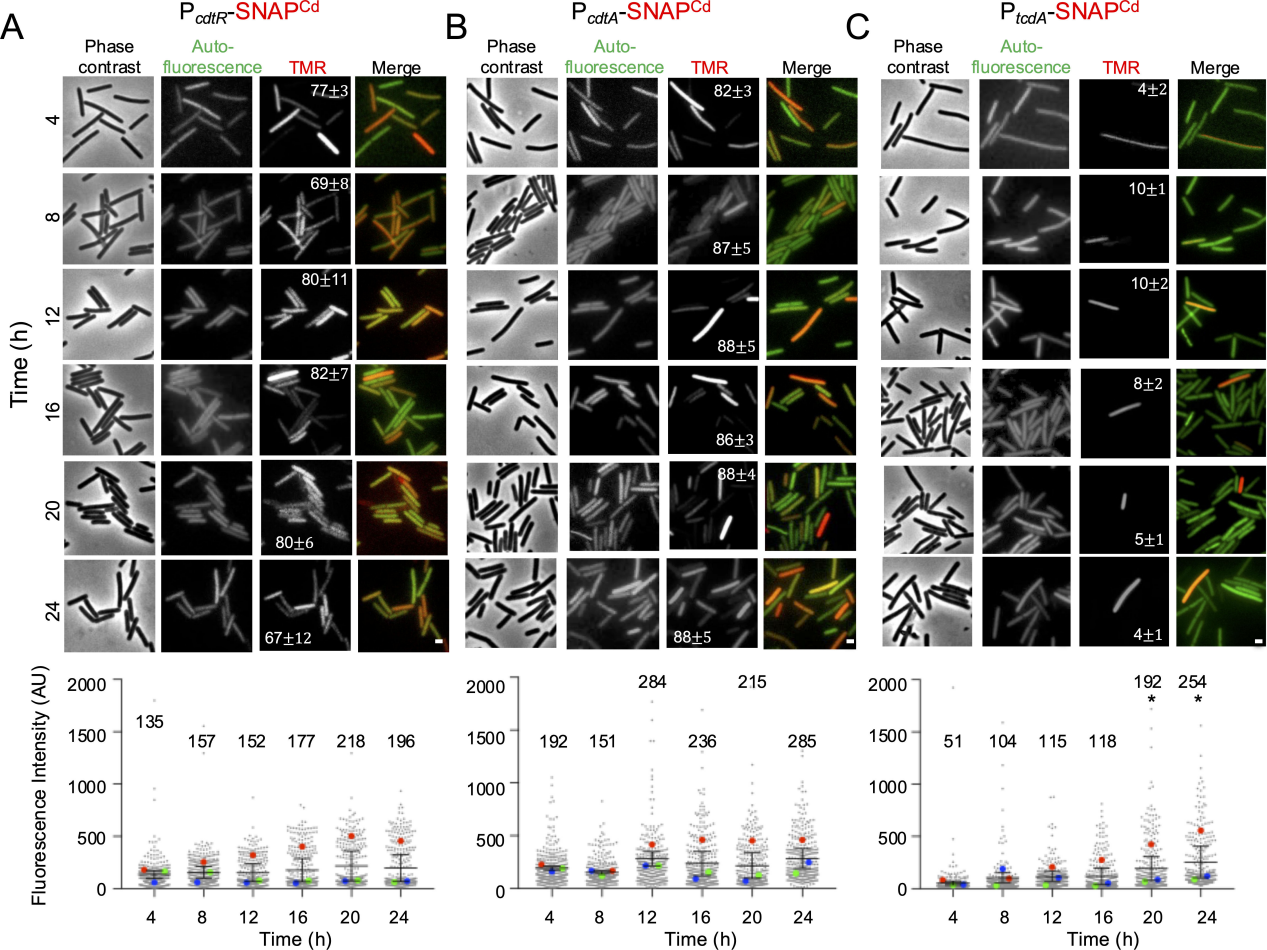
Single-cell analysis of *cdtR*, *cdtA,* and *tcdA* expression. (**A**) Samples were withdrawn from TY cultures of R20291 derivatives carrying the indicated transcriptional fusions of the *cdtR* (**A**), *cdtA* (**B**), or *tcdA* (**C**) promoter regions to the *SNAP^Cd^* reporter. Cells were labeled with the SNAP-tag substrate and imaged by phase contrast (PC) and fluorescence microscopy. The merge corresponds to the auto-fluorescence (AF, green) and TMR images (red). The images are representative of the expression patterns observed. The numbers refer to the percentage ± standard error of the mean (SEM) of cells exhibiting SNAP fluorescence, defined as those with fluorescence intensity exceeding the 99th percentile of the background signal measured in a strain carrying a promoter-less SNAP plasmid (AHCD646; see Fig. S3). At least 100 cells were analyzed. Data shown are from a representative experiment of at least three independent experiments. Scale bar, 1 µm. The bottom panels show the quantification of the intensity of the fluorescence signal resulting from SNAP^Cd^ production in arbitrary units (AU). SuperPlots were used to represent the data from three biological replicates; each dot corresponds to one cell. The large circles represent the means from each experiment, which were used to calculate the mean and standard error of the mean (horizontal lines) for the ensemble of the three experiments. The mean of the fluorescence intensity is shown at the top of each data set. No statistical significance was observed between the data sets shown in panels A and B as determined by a two-way analysis of variance (ANOVA). For panel C (bottom), significant differences at hours 20 and 24 as compared to hour 4 were revealed using ANOVA and Tukey’s test (*P* < 0.05) and are represented by the symbol *.

Expression of the *cdtR* and *cdtAB* genes at the single-cell level has not been reported. We first constructed transcriptional fusions between the *cdtR* and *cdtA* promoters to the *SNAP^Cd^* reporter (36), and for comparison, we also used a P*_tcdA_-SNAP^Cd^* fusion. All transcriptional fusions were inserted into a multicopy plasmid (36). Plasmid copy number variation and/or the increased sensitivity of the plasmid-based reporter, due to its multicopy nature, was previously shown to yield fluorescence intensity distributions similar to those of chromosomally based transcriptional reporters (35). Because of the low percentage of cells with detectable expression of *tcdR* even in strain 630, a P*_tcdR_*-SNAP^Cd^ fusion was not included here. The P*_cdtR_*-, P*_cdtA_*-, and P*_tcdA_*-SNAP^Cd^ fusions were introduced into the R20291 strain of ribotype RT027. Strains carrying P*_tcdA_-SNAP^Cd^*, P*_cdtR_-SNAP^Cd^*, or P*_cdtA_-SNAP^Cd^* fusions were grown in a medium, TY, that supports toxin production (37). To monitor production of the *SNAP^Cd^* reporter, samples of cultures bearing the transcriptional fusions were collected from hour 4 after inoculation, every 4 hours, until 24 hours of incubation (Fig. S2). The cells were labeled with TMR-Star prior to imaging by phase-contrast and fluorescence microscopy. To determine how many cells were in the Toxin-ON state, we set the cutoff for expression at the 99th percentile of the fluorescence intensity measured in a strain carrying a promoter-less SNAP^Cd^ plasmid at a given hour, representing the background (Fig. S3A). In experiments in which the cells bearing the promoter-less SNAP^Cd^ plasmid (labeled with the DNA dye DAPI, yellow arrowheads in Fig. S3B) were mixed with cells expressing a promoter-SNAP^Cd^ fusion (not labeled with DAPI) and imaged in the same field, cells of the first showed a reticular signal in contrast to the uniform signal of the cells expressing the reporter (Fig. S3B; blue arrowheads). Fluorimaging and immunoblot analysis (using an anti-SNAP antibody of whole-cell lysates of the two strains) shows that the TMR-Star substrate does not label any protein in the promoter-less strain (Fig. S3C). Thus, the signal in this strain does not result from SNAP labeling. This allowed us to confirm the cutoff by visual inspection of the images, especially in cells at hours 20–24, labeled with TMR-Star, in which the fluorescence background was higher (Fig. 2; see also Fig. S3B).

SNAP^Cd^ production from P*_cdtR_-SNAP^Cd^* was first detected early during growth and was maintained during stationary phase in almost all cells in the population (~70%–80% of the cells; Fig. 2A). Quantification of the fluorescence signal per cell shows that *cdtR* is homogeneously expressed across the population, although the mean intensity of the signal per cell increases slightly, but not statistically significantly, from hour 12 onward, relative to earlier time points (Fig. 2A). Expression of *cdtA* was detected during growth in 82% ± 3% of the cells, and in at least 86% ± 3% of the cells during stationary phase (Fig. 2B). As for *cdtR*, the intensity of the signal per cell also increased from hour 8 onward (Fig. 2B, bottom panel; average of 151 AU at hour 8 and of 285 AU at hour 24). In contrast, P*_tcdA_-SNAP^Cd^* is expressed in approximately 10% of the cells between hours 8 and 12 and in 4%–8% of the cells during stationary phase, although the mean fluorescence intensity per cell increases at these late time points (Fig. 2C). These observations are in agreement with previous studies showing that *tcdA* expression is bimodal, a pattern thought to result from the *tcdR* positive auto-regulatory loop which is primed by σ^D^ (5, 6) (Fig. 1A). Thus, while the population bifurcates into ON and OFF sub-populations with respect to *tcdA* expression, *cdtR* and *cdtA* show a unimodal expression, with the majority of cells expressing the *cdtR* gene and the *cdtAB* operon.

### *cdtA* and *tcdA* are co-expressed within a small subpopulation of cells

**Fig 3:**
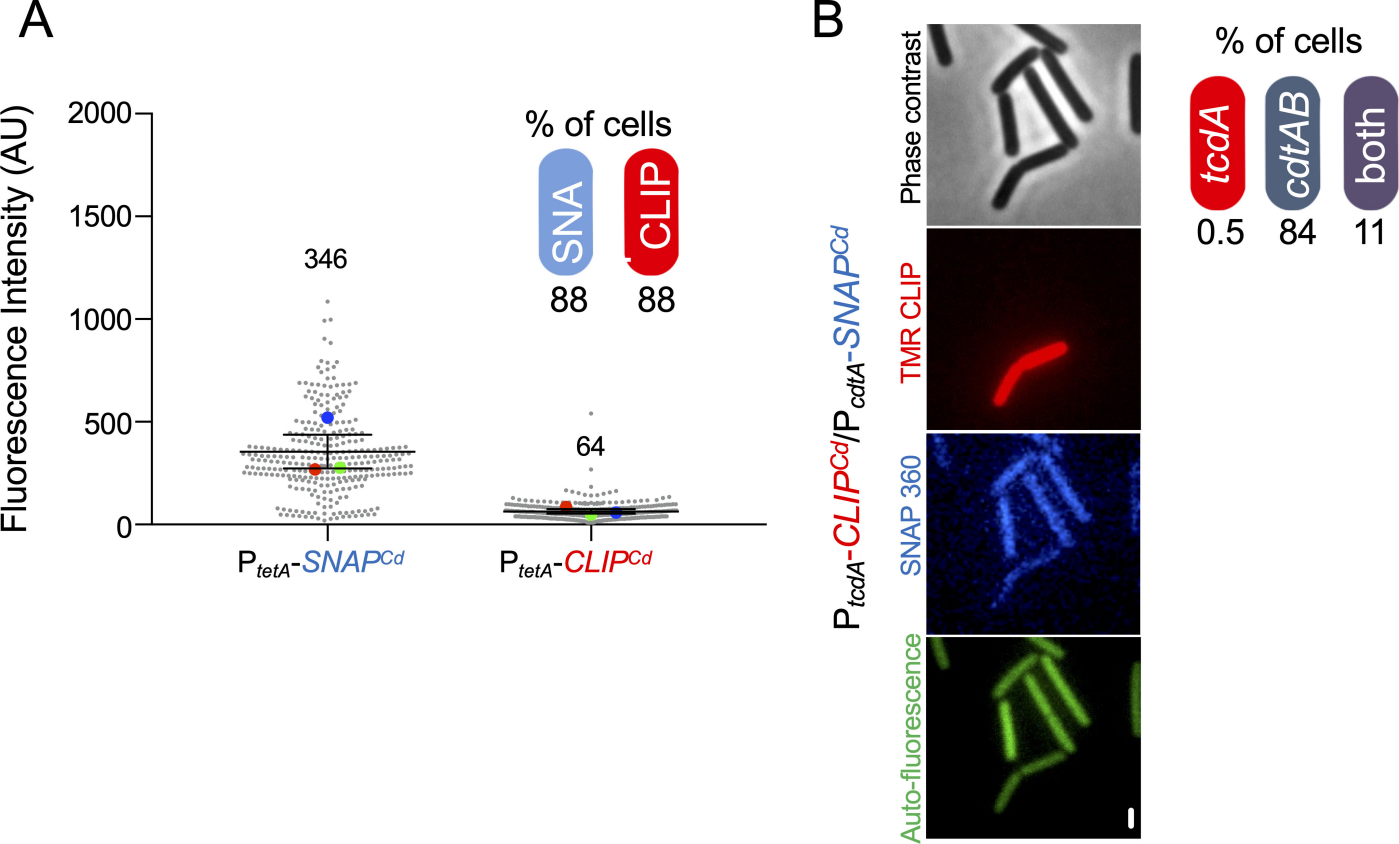
A fraction of the cells expresses both *tcdA* and *cdtA*. (**A**) Fluorescence microscopy analysis of a *C. difficile* strain bearing a fusion of the anhidrotetracycline (ATc)-inducible promoter fused to the *SNAP^Cd^* or *CLIP^Cd^* reporters. Cells were induced with ATc for 1 hour, a sample collected, the cells labeled with the SNAP^Cd^/CLIP^Cd^ substrates, and imaged by phase contrast and fluorescence microscopy to monitor SNAP^Cd^ (SNAP 360 panel) and CLIP^Cd^ (TMR CLIP panel) production. The intensity of the fluorescence signal was quantified (in arbitrary units, AU) from microscopy images. SuperPlots were used to represent the data from three biological replicates (see legend for Fig. 2). The mean of the fluorescence intensity is shown on top of each data set. The percentage of the cells expressing the fusions is shown in the insert. (**B**) Fluorescence microscopy analysis of a R20291 derivative carrying both a P*_cdtA_-SNAP^Cd^* and a P*_tcdA_-CLIP^Cd^* transcriptional fusion. The strain was grown in TY liquid medium until the stationary phase (12 hours), a sample was withdrawn, and the cells were labeled with both the SNAP^Cd^ substrate SNAP-cell 360 and the CLIP^Cd^ substrate TMR-star. SNAP^Cd^ and CLIP^Cd^ production was monitored by fluorescence microscopy. The percentage of the total number of cells that express only one or both fusions is shown on the right panel. Scale bar: 1 µm.

We next addressed the overlap between *tcdA* and *cdtA*-expressing cells. We generated strains carrying pairwise combinations of the *SNAP^Cd^* and *CLIP^Cd^* reporters. We have shown before that the *SNAP^Cd^* and *CLIP^Cd^* reporters allow for orthogonal dual labeling and the simultaneous scoring by fluorescence microscopy of cells expressing the two fusions (38). We first used two strains carrying the anhydrotetracycline (ATc) responsive P*_tetA_* promoter (39) fused to the *SNAP^Cd^* or the *CLIP^Cd^* reporters in a multicopy plasmid and quantified the fluorescence signal in fields of vegetative cells bearing either fusion, after induction with ATc and labeling with the fluorescent TMR-Star substrate for CLIP^Cd^ and SNAP-cell 360 substrate for SNAP^Cd^ (Fig. 3A). The average signal intensity was 346 AU for the P*_tet_*-SNAP^Cd^ fusion and 64 AU for P*_tet_*-CLIP^Cd^, but the number of cells showing fluorescence signal was similar for both reporter fusions (88%; insert in Fig. 3A). This indicates that the percentage of cells expressing *tcdA* and *cdtA* may be calculated using this dual labeling system.

An R20291 derivative carrying both P*_tcdA_-CLIP^Cd^* and P*_cdtA_-SNAP^Cd^* transcriptional fusions in a multicopy plasmid was then grown in TY medium for 12 hours. To monitor production of the SNAP^Cd^ and CLIP^Cd^ tags, samples withdrawn from cultures were labeled with TMR-Star and SNAP-cell 360 prior to imaging by phase-contrast and fluorescence microscopy (Fig. 3B). We found cells that expressed only *tcdA* (0.5%) or only *cdtA* (84%), but most of the *tcdA* ON cells were contained within the *cdtA* ON population (11%) (Fig. 3B). We infer that most of the TcdA producer cells are also CDT producers, and that CDT production occurs independently of TcdA in most of the cells in the population.

### CdtR regulates *cdtA* and *tcdA* expression but not its own

CdtR is a response regulator of the LytTR family, previously shown to regulate *cdtA* and *tcdA* expression (23, 30–32). Using the Phyre2 server (40), we found that the closest structural homolog of CdtR is ComE, a response regulator of the LytTR family involved in the development of competence in *Streptococcus pneumoniae* (Fig. S1). ComE is activated by phosphorylation of a conserved aspartate residue (D58) (41; Fig. S1). The homology model confirms the previous suggestion, made on the basis of sequence alignments, that D61 is the phosphoryl-acceptor residue in CdtR (30; Fig. S1). Replacement of D61 by Ala inactivated CdtR, whereas the phosphomimetic substitution D61E partially complemented a Δ*cdtR* mutant for CDT production and cytotoxicity (30).

**Fig 4:**
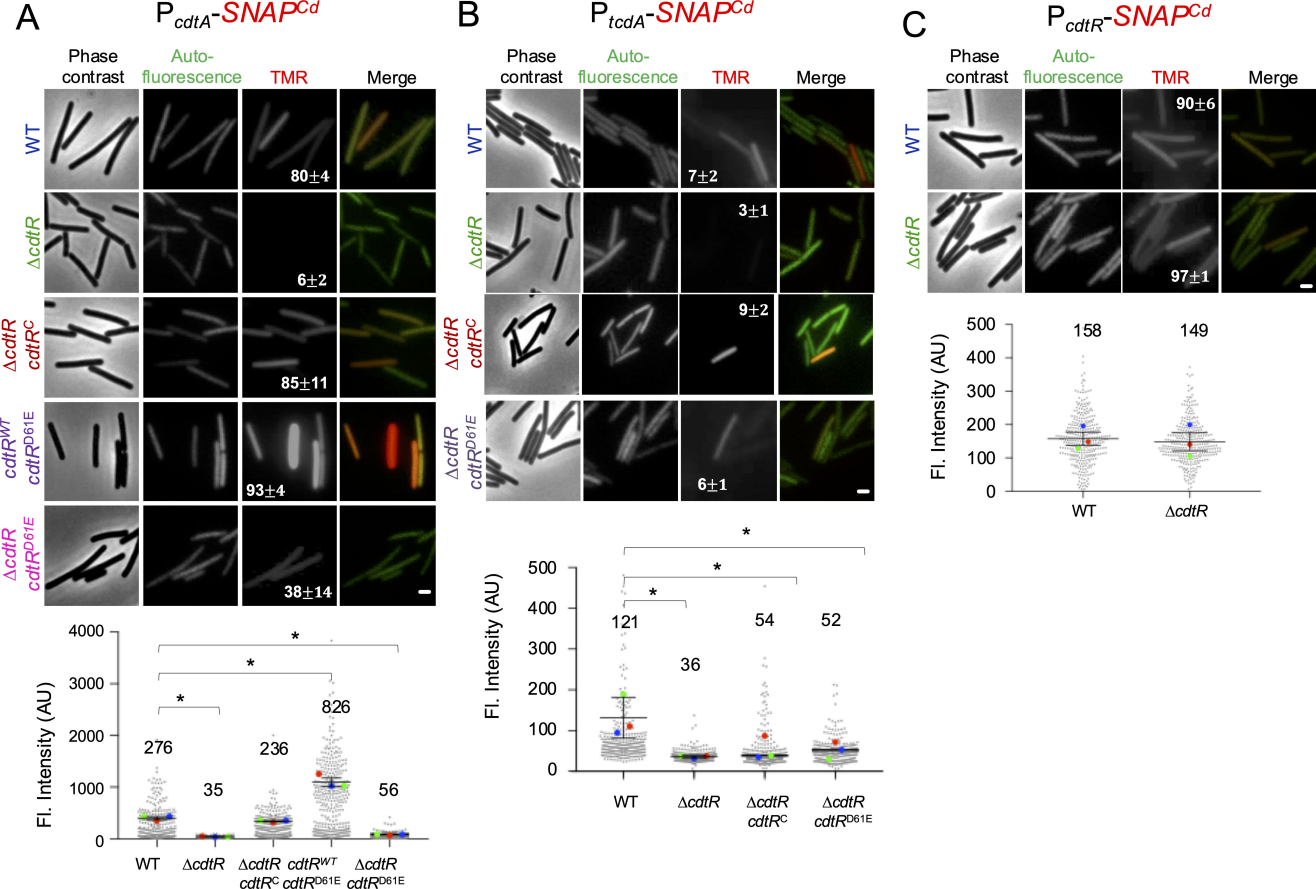
CdtR regulates *cdtA* and *tcdA* expression but not its own. Fluorescence microscopy analysis of *C. difficile* R20291 WT and *cdtR* mutant strains carrying the indicated *cdtR* alleles bearing a replicative plasmid containing the *cdtA* ( **A**), *tcdA* ( **B**), and *cdtR* ( **C**) promoter fused to the *SNAP^Cd^* reporter. Cells were grown in TY liquid medium, collected at mid-log, and labeled with the SNAP^Cd^-tag substrate. Scoring of the vegetative cells expressing the transcriptional fusion is shown in the percentage ± SEM in the TMR panel, as defined previously (see Fig. 2). At least 100 cells were analyzed. Scale bar: 1 µm. Bottom panels: following examination by PC and fluorescence microscopy to monitor SNAP^Cd^ production, the signal per cell was quantified in AU. The mean of the fluorescence intensity is shown on top of each graph. SuperPlots were used to represent the data from three biological replicates (see Fig. 2). Relevant significant differences as compared to the WT strain, revealed using ANOVA and Tukey’s test (*P* < 0.05), are represented by the symbol * in panel A and B. Using Student’s *t*-test (*P* < 0.05), there were no significant differences between strains in panel C (bottom).

To examine the role of D61 on the expression of *cdtA* at the single-cell level, we first constructed a *cdtR* in-frame deletion mutant (Fig. S4A). Then, we re-created the phosphomimetic D61E allele and used it to complement the Δ*cdtR* mutation in a single copy at the *pyrE* locus. Finally, we introduced the P*_cdtR_-SNAP^Cd^*, P*_cdtA_-SNAP^Cd^*, or P*_tcdA_-SNAP^Cd^* fusions into these strains. To monitor production of the SNAP-tag, samples of cultures bearing the transcriptional fusions grown in TY medium were collected at midlog, and cells were labeled with TMR-Star and imaged by phase-contrast and fluorescence microscopy (Fig. 4). In line with previous studies, expression from P*_cdtA_-SNAP^Cd^* and P*_tcdA_-SNAP^Cd^* is markedly reduced in the Δ*cdtR* mutant, detected in only ~6% and 3% of cells, with mean fluorescence intensities of 35 AU and 36 AU, respectively—compared to 276 AU and 121 AU in a wild-type background (Fig. 4A and B) (23, 31). The percentage of cells expressing P*_cdtA_-SNAP^Cd^* or P*_tcdA_-SNAP^Cd^* was restored when Δ*cdtR* was complemented with a wild-type copy of the gene at the *pyrE* locus. The average intensity of the fluorescence signal per cell was not far from the WT in the case of P*_cdtA_-SNAP^Cd^* (236 AU as compared to 276 AU; Fig. 4A). However, in the case of P*_tcdA_-SNAP^Cd^*, although the average fluorescence intensity per cell increases, it does not reach wild-type levels (54 AU as compared with 121 AU for the WT; Fig. 4B). Complementation of the *cdtR* deletion mutation with the *cdtR^D61E^* allele partially restored P*_cdtA_-SNAP^Cd^* expression (to 38% ± 14% of the cells as compared to 80% ± 4% for the WT; Fig. 4A) and also P*_tcdA_-SNAP^Cd^* expression (6% ± 1% of cells) (Fig. 4B). The average signal per cell for both transcriptional fusions, however, was significantly lower than for the WT (56 AU as compared to 276 AU for P*_cdtA_-SNAP^Cd^* and 52 AU as compared to 121 AU for P*_tcdA_-SNAP^Cd^*; Fig. 4A). Complementation of Δ*cdtR* at *pyrE* results in higher expression of the gene compared to the normal chromosomal locus and thus the numbers probably overestimate the activity of CdtR^D61E^ (30). Strikingly, in a merodiploid strain, in which both the wild-type and the *cdtR^D61E^* allele at *pyrE* are present, the expression of P*_cdtA_-SNAP^Cd^* is highly increased: 93% ± 4% of the cells show fluorescence and the average intensity of the fluorescence signal is 826 AU as compared to 276 AU for the WT (Fig. 4A). Previous studies have noted that strains with multicopy alleles of *cdtR* showed increased CDT production (32). The results suggest that the cellular concentration of CdtR is normally sufficient to activate *cdtA* expression in most cells, but is set to achieve a certain level of *cdtA* expression and CDT production. Presumably, the total cellular concentration and activity of CdtR/CdtR^D61E^ go above this level (also see Discussion).

A *cdtR* deletion had no impact on P*_cdtR_-SNAP^Cd^* expression (Fig. 4C), indicating that CdtR is not auto-regulatory, possibly explaining why the expression of *cdtA* is unimodal.

### TcdR affects *cdtA* expression

**Fig 5:**
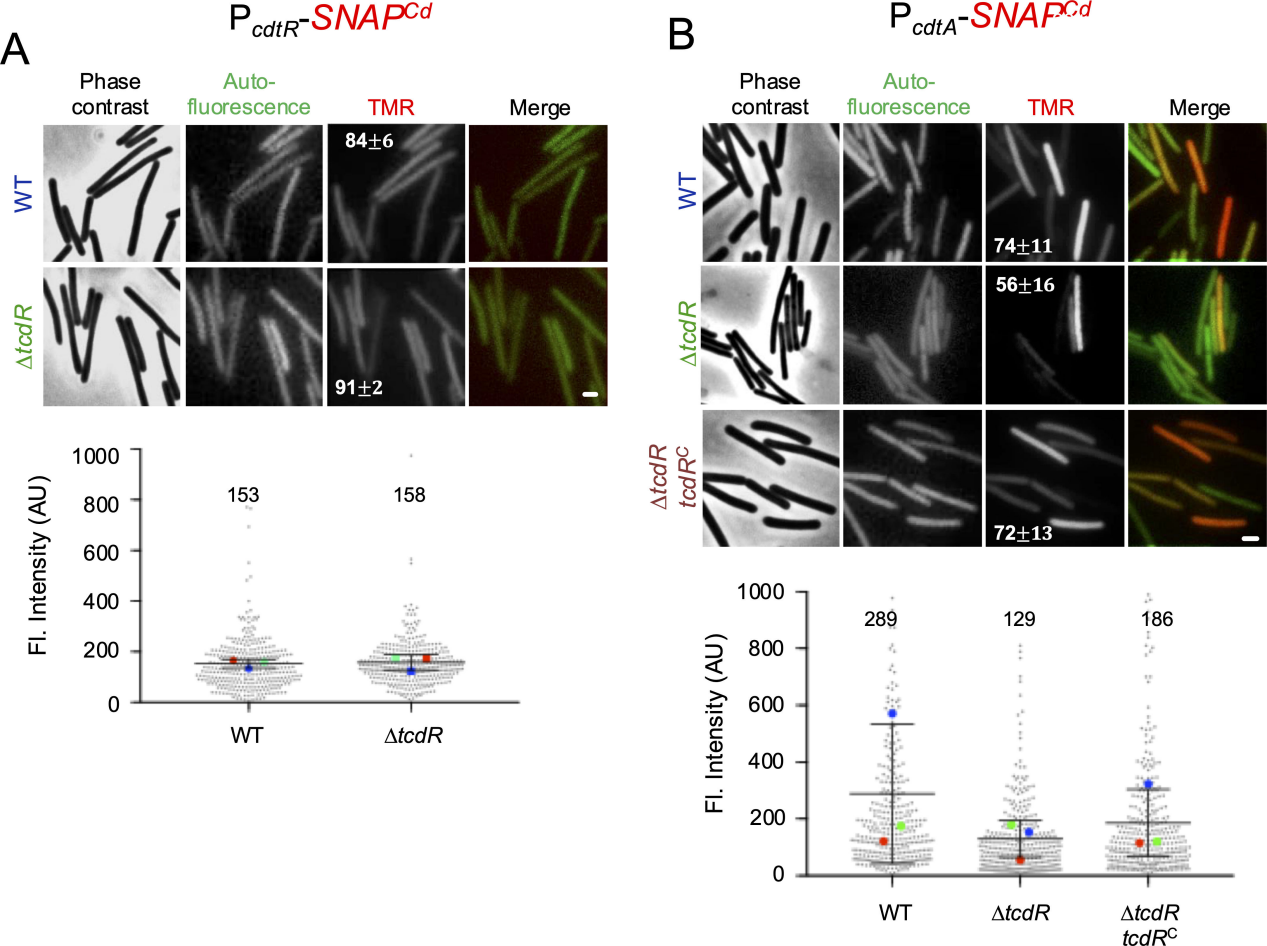
TcdR influences *cdtA* but not *cdtR* expression. (**A and B**) Fluorescence microscopy analysis of *C. difficile* R20291 WT, *tcdR* mutant, and the complemented strain (*tcdR*^C^). These strains contained a replicative plasmid with *cdtR* (**A**) or *cdtA* (**B**) promoters fused to the *SNAP^Cd^* reporter. Cells were grown in TY liquid medium, collected at mid-log, and labeled with the SNAP^Cd^-tag substrate. Scoring of the vegetative cells expressing the transcriptional fusions is shown in percentage ± SEM in the TMR panel, as defined previously (see Fig. 2). At least 100 cells were analyzed. Scale bar: 1 µm. Bottom panels: the cells were imaged by PC and fluorescence microscopy to monitor SNAP^Cd^ production, and the signals for the *cdtR* (**A**) and *cdtA* (**B**) strains were quantified in AU. The mean of the fluorescence intensity is shown on top of each graph. SuperPlots were used to represent the data from three biological replicates (see Fig. 2). Using Student’s *t*-test (*P* < 0.05), there were no significant differences in panel A; in panel B, no significant differences were seen when the data were analyzed using ANOVA and Tukey’s test.

CdtR impacts the regulation of TcdA/TcdB production from the PaLoc (32). On the other hand, RT078 strains produce CDT but do not code for a functional CdtR (30, 32). We wanted to test whether TcdR could also influence the expression of *cdtA*. To test this possibility, an in-frame deletion of *tcdR* was constructed in R20291 using ACE (Fig. S4B). The P*_cdtR_-SNAP^Cd^* and P*_cdtA_-SNAP^Cd^* fusions were introduced in this mutant, cultures were grown in TY medium, samples collected at mid log, the cells were labeled with TMR-Star, and imaged by phase-contrast and fluorescence microscopy (Fig. 5). Neither the number of cells expressing P*_cdtR_-SNAP^Cd^* nor the average fluorescence intensity per cell was affected in the absence of TcdR (Fig. 5A). In contrast, the percentage of cells expressing P*_cdtA_-SNAP^Cd^* was reduced from 74% ± 11% in the WT to 56% ± 16% in the mutant in the *tcdR* mutant (Fig. 5B), and although not statistically significant, the average fluorescence intensity per cell decreased to 129 AU as compared to 289 AU for the WT (Fig. 5B). P*_cdtA_-SNAP^Cd^* expression was restored when Δ*tcdR* was complemented with a wild-type copy at the *pyrE* locus (Fig. 5B). Thus, during growth, TcdR, directly or indirectly, influences *cdtA* expression.

### CdtA is secreted during growth

**Fig 6:**
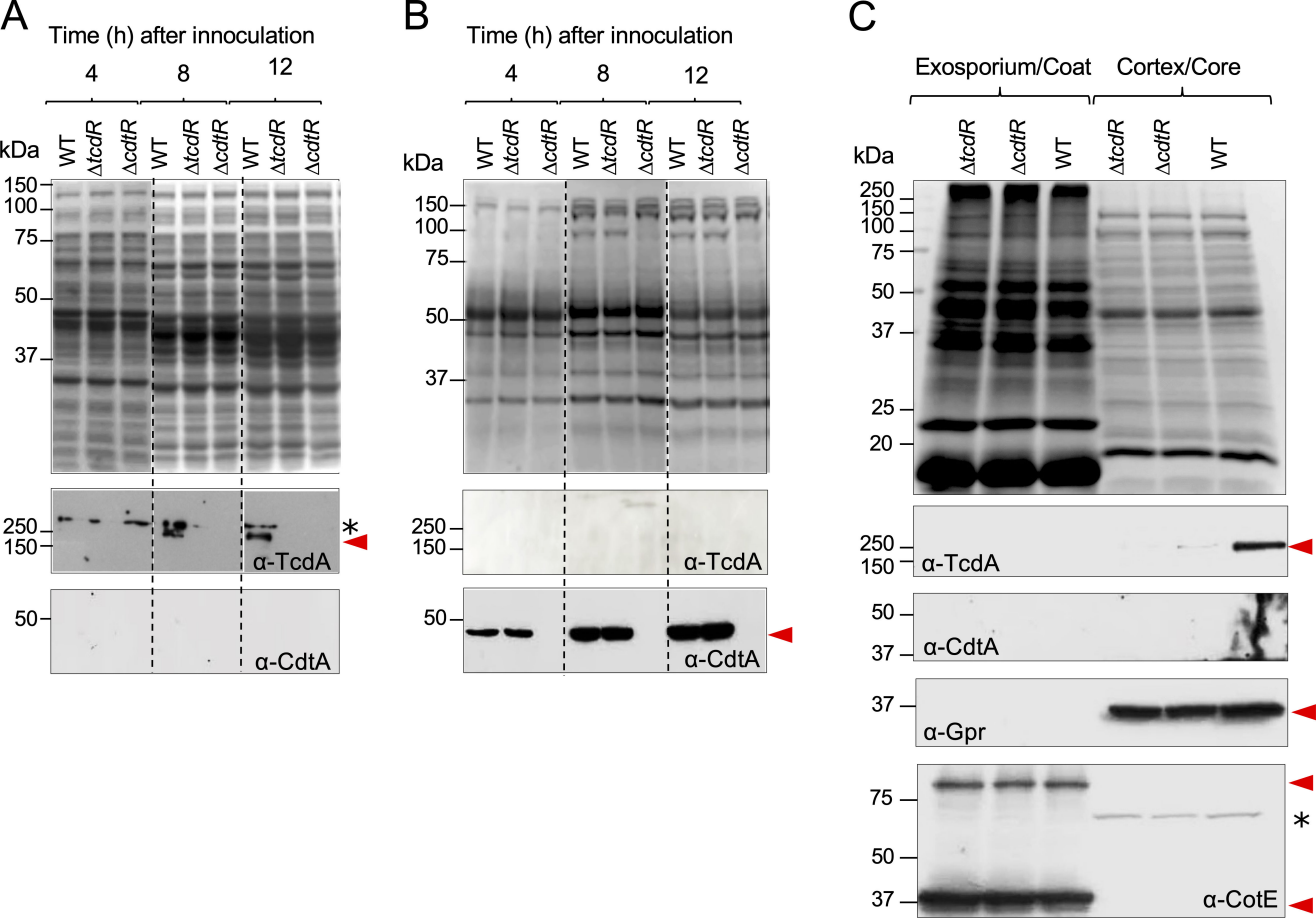
CdtA and TcdA accumulation. R20291 and congenic *tcdR* and *cdtR* mutants were grown in TY liquid medium and collected at different time points (4, 8, and 12 hours). Cell extracts were obtained by cell lysis (**A**), and the supernatants were 100× concentrated (**B**). Samples were resolved by SDS-PAGE and stained with Coomassie brilliant blue for total protein content analyses (upper panel). The immunoblot analysis is represented in the bottom panels, in which TcdA and CdtA were detected using anti-TcdA or anti-CdtA antibodies. The position of molecular weight markers (in KDa) is shown on the left side of the panel. Dashed lines in panels A and B indicate where the gel and western blot were cropped. An asterisk in panel A shows the position of a cross-reactive species. (**C**) Spores formed by R20291 (WT) and the *tcdR* and *cdtR* mutants were purified and fractionated into a cortex/coat/exosporium and a cortex/core layer. Proteins were extracted, resolved by SDS-PAGE, and stained with Coomassie Brilliant Blue (top panel). Immunoblot analyses are shown in the bottom panels with the indicated antibodies: anti-TcdA and anti-CdtA were used to detect the toxins, while anti-Gpr and anti-CotE were used as fractionation controls for the most internal and the most external layers, respectively. The position of molecular weight markers (in KDa) is shown on the left side of the panel. The asterisk shows the position of a cross-reactive species.

In order to independently test the results obtained by single-cell analysis of the transcriptional fusions to the *SNAP^Cd^* reporter, we used immunoblot analysis to monitor TcdA and CdtA accumulation over time in the WT and in the *tcdR* and *cdtR* mutants. Cells were grown in TY medium, and cells and supernatants were collected after 4, 8, and 12 hours after inoculation. TcdA was only detected in cell extracts at hours 8 and 12, and CdtA only in the supernatant, but from hour 4 (Fig. 6A and B). No TcdA signal was detected in the *tcdR* or *cdtR* mutants, as described before (3, 32). In contrast, CdtA accumulation was only dependent on CdtR (Fig. 6A and B). Thus, CdtA starts to accumulate during vegetative growth, and although we detected an effect of TcdR on *cdtA* expression (Fig. 5B), this effect does not have an impact on protein accumulation. Moreover, TcdA seems to remain mostly associated with cells, at least under our conditions, while CdtA is secreted (Fig. 6A and B) (28). The results suggest that CdtA may act before TcdA, in line with the idea that CDT is important for adhesion to epithelial cells (24). The results are also in agreement with the single-cell analysis of P*_cdtA_-SNAP*^Cd^ and P*_tcdA_-SNAP*^Cd^ expression (Fig. 2).

### CDT does not associate with mature spores

Previous results in our laboratory showed that TcdA associates with the surface of mature spores (6). To test whether CDT is also associated with spores, we purified spores from the WT and *cdtR* and *tcdR* mutants, and these were fractionated into a cortex/coat/exosporium and a core/cortex fraction (6). We then used immunoblotting to check for the presence of TcdA and CdtA in these fractions. In contrast to strain 630, where TcdA is detected in the two fractions, in R20291, accumulation of TcdA is only associated with the core/cortex fraction, and no or reduced levels of toxin were detected in *tcdR* or *cdtR* mutant spores, respectively (6; Fig. 6C). We did not detect CdtA in any of the fractions obtained from purified spores (Fig. 6C).

## DISCUSSION

### Expression of the toxin-encoding genes in strain R20291

Single-cell analysis is of critical importance in revealing population heterogeneity and in identifying specific subpopulations. Here, we used single-cell analysis to unveil details of toxin gene expression in strain R20291. We show that a small population of cells expresses *tcdA* (~10%), while almost all cells express *cdtA* (~90%). It is tempting to speculate that this population structure underpins a cooperative behavior with respect to the production of the PaLoc- and CdtLoc-encoded toxins. The two populations overlap partially; however, most of the *tcdA* ON cells are contained within the *cdtA* ON population. While *cdtA* transcription is detected during growth, *tcdA* transcription is mainly induced upon entry into the stationary phase of growth. In parallel, CDT is secreted and detected in the culture supernatant during growth, whereas TcdA remains mostly associated with the cells. Thus, expression of *cdtA* and secretion of CDT precede the expression of *tcdA*.

The low percentage of cells expressing *tcdA* in our study is in agreement with previous studies showing that the fraction of cells expressing *tcdA* in R20291 is reduced relative to strain 630Δ*erm* (5). This low percentage reflects the lower amount of σ^D^ production in R20291 when compared with 630Δ*erm*. This is due to a regulatory switch that controls expression of the flagellar regulon, including *sigD*, which codes for σ^D^. In R20291, this “flagellar switch” swings between the ON and OFF state during growth, while in strain 630Δ*erm,* it is locked in the ON orientation, leading to increased flagellar gene expression (42, 43).

Rybicki et al. suggested that pathogens with locally acting toxins (the case of TcdA) have smaller infective doses than pathogens with highly diffusive toxins. When the toxin is highly diffusive, the initial pathogen population grows and establishes only if the initial dose is sufficiently high or the pathogen is not eliminated by the initial immune response. In contrast, with low diffusion, the pathogen can grow even if the initial dose is small (44). This can be seen as a “stealth attack strategy” (45), as localized mechanisms may lower the chances of the immune system detecting the pathogen.

### Cross-regulation between the PaLoc and the CdtLoc

We confirmed that CdtR is required for *cdtA* and *tcdA* expression in R20291. Interestingly, and in contrast to what happens with other response regulators of the LytTR family, which are under positive auto-regulation, CdtR does not control its own expression (46). The lack of a positive auto-regulatory loop involving CdtR may explain why *cdtA* does not present a bimodal pattern of expression. Bilverstone et al., who previously proposed that CdtR is activated by phosphorylation at D61 within the receiver domain of the response regulator, showed that the phosphomimetic substitution CdtR^D61E^ supports only about 40% of CDT activity (30). In line with these observations, we show that the D61E form of CdtR allows only partial restoration of *cdtA* or *tcdA* expression. However, when two copies of *cdtR* are present in the cell, the WT and the *cdtR^D61E^* allele, the expression of *cdtA* is significantly increased. In many response regulators, phosphorylation promotes dimerization and allows the stable recognition of the DNA binding sites. In ComE, a non-phosphorylatable form, D58A, is mostly a monomer in solution and fails to activate expression from the target *comCDE* operon, whereas the phosphomimetic D58E variant, which allows for constitutive competence, is a dimer even in the absence of DNA (34). Dimerization occurs via interactions of the REC domains, which are reinforced by their phosphorylation (34). In the dimer, the LytR domains are arranged in tandem, consistent with binding to two direct repeats present in the target genes (34). The binding sites for CdtR are not yet known. However, the residues in the LytR domain of *Staphylococcus aureus* AgrA (47) that make sequence-specific contacts with two consecutive major grooves in the DNA (H169 and R233) essential for DNA-binding activity, as well as a residue that contacts the intervening minor groove (N201), are conserved in CdtR (H173, R239, and K205; Fig. S1). This suggests a similar mode of DNA binding for the LytTR family members (48, 49). Also, although likely, we do not yet know whether phosphorylation leads to dimerization of CdtR. One possibility is that maximal expression of *cdtA* may normally take place at a certain level of phosphorylated CdtR, and that accumulation of active CdtR past this level may allow binding to low-affinity sites, resulting in repression. The shutdown of *comCDE* expression was proposed to result from the continued accumulation of ComE, which would out-compete the phosphorylated form (50, 51). An alternative model, however, is that in the WT/D61E strain, the overall concentration of CdtR is just higher, and the heterodimer is functional.

The kinase that phosphorylates CdtR is presently unknown. *cdtR*, however, is adjacent to the gene coding for a sensor histidine kinase, CDR20291_2490, and this gene is just upstream of CDR20291_2488, coding for another LytR family response regulator (Fig. 1B; Fig. S1A). Whether the CDR20291_2490 kinase phosphorylates CdtR at D61 remains to be tested. Interestingly, ComE is phosphorylated at two sites: at D58 by the ComD histidine kinase and at T128 by the StkP serine/threonine kinase (52). T128 phosphorylation is required for ComE activation in specific environmental conditions. It seems unlikely that CdtR is phosphorylated at a homologous position because the T residue is not conserved (Fig. S1A). *cdtA*, but not *cdtR* expression, is reduced in a *tcdR* mutant, supporting the idea of cross-regulation within the two loci. Possibly, RT078 strains (TcdA^+^TcdB^+^CDT^+^), which have a non-functional CdtR (30), produce CDT in a TcdR-dependent manner (32).

### Spores do not carry CDT

**Fig 7:**
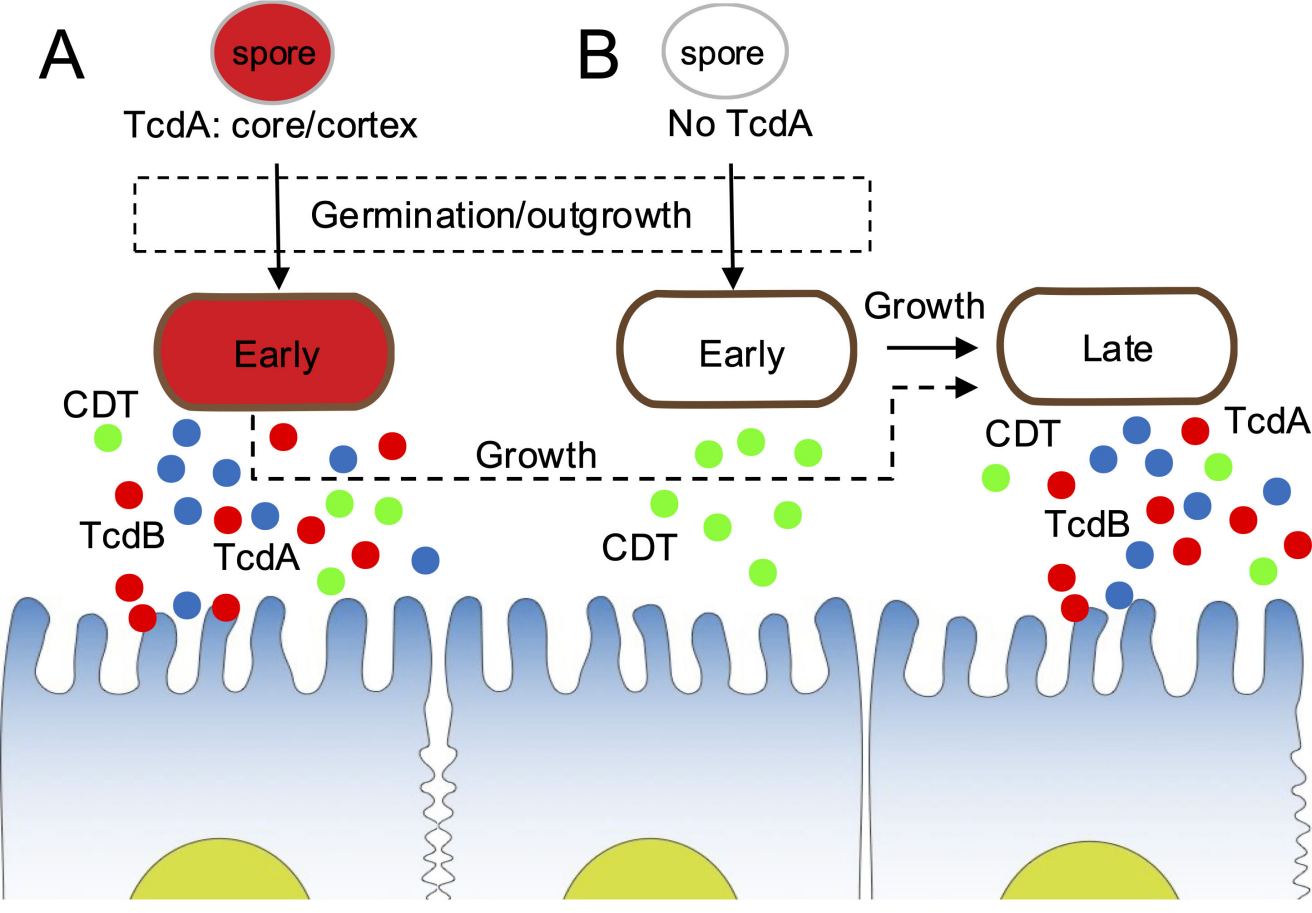
Model for the temporal relationship between TcdA/B and CDT. The figure represents spores produced by a strain of ribotype 027, such as R20291, that produce TcdA/B in the forespore and accumulate the toxins in the core/cortex compartments of spores (**A**) (6). A fraction of the sporulating cells will not produce TcdA/B, and their spores will not carry the toxins (**B**). For the spores in A, germination may result in cells able to immediately release TcdA/B and to produce and secrete CDT. Growth of these cells will result in continued production of CDT and the production of TcdA/B upon entry into the stationary phase. Thus, TcdA/B and CDT may cooperate during the initial stages of infection and act mostly sequentially during growth/entry into the stationary phase, even though the sub-population of CDT- and TcdA/B-producers partially overlaps. For those spores that do not carry TcdA/B (**B**), CDT is released from the vegetative cells soon after germination, and TcdA/B, presumably produced in a cell density-dependent manner, will only act later.

We have shown recently that TcdA/TcdB are produced by a fraction of sporulating cells in a culture and that the toxins associate with the spores produced by those cells (6). Spores are cytopathic, and we have suggested that they act as toxin delivery vehicles (6). While in other strains, the TcdA/TcdB are produced in the mother cell or in the forespore, in an RT027 strain such as R20291, the toxins are specifically produced in the forespore and associate with the spore cortex or core layers (6). We have suggested that in this case, the toxins could be released from the cells soon after germination (6). The toxins thus released may have an immediate effect on the host cells, at least in part coincident with CDT production and secretion (Fig. 7). These cells will then produce TcdA/B at the end of growth. Thus, even though production of CDT and TcdA/TcdB is largely segregated temporally, they may cooperate during the early stages in infection and work sequentially later, with part of the populations of CDT- and TcdA/B-producers partially overlapping. For those spores that do not carry TcdA/TcdB, the cells resulting from germination first produce and secrete CDT, with TcdA and TcdB production being activated upon entry into the stationary phase. The temporal separation between CDT and TcdA/B production and release may be crucial for facilitating proper cell adhesion and epithelium colonization before the immune system can mount a response. CDT not only suppresses a protective host eosinophilic response (25) but also helps bacteria to adhere to epithelial cells (24) while inducing the formation of biofilm-like microcolonies (27). Understanding the timings/level of expression and toxin production *in vitro* is crucial before designing experiments in animal models to further investigate the function of and interplay between the CDT and TcdA/B toxins.

## MATERIALS AND METHODS

### Growth conditions and general methods

Bacterial strains and their relevant features are listed in Table S1. *Escherichia coli* strain DH5α (Invitrogen) was used for molecular cloning, while strain HB101 (RP4) was used for plasmid transfer into *C. difficile* by conjugation (53). Both strains were grown in Luria-Bertani medium with ampicillin (100 µg/mL) or chloramphenicol (20 µg/mL) as required. The *C. difficile* strains used are congenic derivatives of strain R20291Δ*pyrE* (54) and were routinely grown anaerobically (5% H_2_, 15% CO_2_, 80% N_2_) at 37°C. Brain heart infusion (BHI) medium (Oxoid) was used for growth and maintenance of *C. difficile* strains supplemented with cefoxitin (25 µg/mL) and/or thiamphenicol (15 µg/mL) when required. For toxin assays, tryptone yeast extract (30 g/L tryptone; 20 g/L yeast extract) (TY) was used. For the selection of recombinants, a minimal medium (CDMM) (55) with 1% agar containing uracil (5 µg/mL) and 5-fluoroorotic acid (5-FOA: 2 mg/mL) was used. For gene complementation and *pyrE* reversion, none of these supplements were added to CDMM. All the primers used in this study are listed in Table S2, and all the plasmids used or constructed during this study are listed in Table S3.

### Fluorescence microscopy and image analysis

For SNAP^Cd^ labeling, 200 µL samples of *C. difficile* cultures were mixed with 250 nM SNAP-cell TMR‐Star substrate (New England Biolabs) for 30 minutes, in the dark. For dual labeling assays, 200 µL samples were labeled with 250 nM of the CLIP-tag cell-permeable substrate TMR-star (New England Biolabs) for 30 minutes and with 250 nM SNAP-cell 360 (New England Biolabs) during the last 15 minutes of incubation with the first substrate. After labeling, cells were collected by centrifugation (4,000 × *g*, for 5 minutes) and washed twice with 1 mL of PBS (137 mM NaCl, 10 mM phosphate, 2.7 mM KCl, pH 7.4). The washed cell sediment was resuspended in 20 µL of PBS, and 4 µL were mounted on 1.7% agarose-coated glass slides. Microscopy images were acquired using a Leica DM6000B upright microscope equipped with an Andor iXon 885 EMCCD camera and controlled with the MetaMorph V5.8 software. The images were acquired with a 100× 1.4 NA immersion objective and a 1.6× optovar, the fluorescence filter sets Cy3 and FITC, and phase contrast optics.

Cell segmentation was conducted using Cellpose (version 3.1.1.1), a generalist deep learning-based algorithm for cellular segmentation (56). The software was operated via its Python-based graphical user interface. The estimated cell diameter was set to 30 pixels, based on empirical measurements and Cellpose’s automatic estimation. Input images were processed in grayscale mode. Quantitative image analysis, including pixel-based measurements and downstream processing, was conducted using Fiji/ImageJ (57).

### Immunoblot analysis of cell extracts and culture supernatants

Twenty milliliters of *C. difficile* cultures was centrifuged for 10 minutes at 4,000 × *g* (4^o^C) after specific time points. Total cell extracts were obtained from the cell sediment, which was lysed in a French pressure cell (18,000 lb/in^2^). Protein concentration in supernatants or cell sediments was determined with the Bradford Protein Assay (Bio-Rad, Hercules, CA). Supernatants were concentrated 100 times using Amicon centrifugal filter units (Sigma-Aldrich). Samples of 5 µg (cell sediment) or 10 µg (supernatant) were resolved by 12.5% SDS-PAGE. Gels were stained with Coomassie Brilliant Blue R-250, or subject to immunoblot analysis with an anti-TcdA antibody at a dilution of 1:5,000 (from Santa Cruz Biotechnology), an anti-CdtA antibody at a dilution of 1:5,000 (from List Biological Laboratories), or an anti-SNAP antibody at a dilution of 1:1,000 (from New England Biolabs). The following secondary antibodies conjugated to horseradish peroxidase were used: anti-mouse 1:2,000, anti-chicken 1:2,000, and anti-rabbit 1:5,000. The immunoblots were developed with enhanced chemiluminescence reagents (Amersham Pharmacia Biotech).

### Spore production, purification, and fractionation

For spore production, 1.5 mL of an overnight *C. difficile* culture was inoculated into 150 mL of liquid BHI and incubated at 37°C in anaerobic conditions for 7 days. The cell sediments, which were collected by centrifugation at 4,800 × *g* for 10 minutes, were resuspended and kept in cold water (4°C) for 48 hours. For spore purification, the cold suspension was centrifuged again as above, and the cells' sediment was resuspended in PBS with 0.1% Tween-20 before being added to a 42% Renografin (Bayer) step gradient (58). After a 20 minute centrifugation at 4,800 × *g*, the sediment was washed twice with PBS with 0.1% Tween-20 and twice in water. The final spore suspensions were stored at 4°C until further use. For spore fractionation, the spore coat/exosporium was separated from the spore cortex/core. Spores were resuspended in 50 µL of decoating buffer (10% glycerol, 4% SDS, 10% β-mercaptoethanol, 1 mM DTT, 250 mM Tris, pH 6.8) and boiled for 5 minutes. After centrifugation, the supernatant, which comprises the coat/exosporium content, was separated from the sediment, mainly the cortex/core. The material in this fraction was washed twice with PBS with 0.1% Tween-20 and incubated with 50 mM Tris-HCl, pH 8.0, and 2 mg/mL lysozyme for 2 hours at 37°C. Gels and immunoblots were as above, except that additionally, an anti-Gpr antibody was used at a dilution of 1:10,000 (59) and an anti-CotE (59) was used at a dilution of 1:1,000. The following secondary antibodies conjugated to horseradish peroxidase were used, as follows: anti-mouse 1:2,000, anti-chicken 1:2,000, and anti-rabbit 1:10,000. The immunoblots were developed as described above.

### Additional methods

Methods for mutant construction through allelic exchange in *C. difficile* (60*)*, the construction of *SNAP^Cd^* and *CLIP^Cd^* transcriptional fusions, and AlphaFold2 modeling (61, 62, 63) are given in the supplemental material.

## SUPPLEMENTAL MATERIAL

Supplemental Material msphere.00186-25-s0001.pdf Supplemental text, figures, and tables.

ASM does not own the copyrights to Supplemental Material that may be linked to, or accessed through, an article. The authors have granted ASM a non-exclusive, world-wide license to publish the Supplemental Material files. Please contact the corresponding author directly for reuse.
