# Supplementary material for: Temporally segregated subpopulations of CDT and TcdA producer cells of Clostridioides difficile
Source: mSphere. 2025 Sep 3;10(9):e00186-25. doi: 10.1128/msphere.00186-25 (PMC12482169; doi:10.1128/msphere.00186-25)
Supplement: Supplemental Material — Supplemental text, figures, and tables. [file msphere.00186-25-s0001.pdf]

SUPPLEMENTARY MATERIAL

Temporally segregated subpopulations of CDT and TcdA producer cells of *Clostridioides difficile*

Sara Ramalhete[1#], Isabel Roseiro[1], Carolina P. Cassona[1#], Carolina Alves Feliciano[1], Mónica Serrano[1*] and Adriano O. Henriques[1*]

[1]Instituto de Tecnologia Química e Biológica António Xavier, Avenida da República, 2780-157 Oeiras, Portugal;

#Present address: Instituto de Biologia Experimental e Tecnológica, Avenida da República, 2780-157 Oeiras, Portugal

*Co-corresponding authors:

Phone: +351 21 4469521; *Fax*: +351 21 441 1277; *Email*: aoh@itqb.unl.pt *or* serrano@itqb.unl.pt

Running title: Single cell analysis of *cdtAB* and *tcdA* transcription

Supplementary Material and Methods

***SNAP^Cd^* and *CLIP^Cd^* transcriptional fusions.** *SNAP^{Cd}* was PCR-amplified from pFT47 (36) using the primer pair SNAP-SOE-Fw/ SNAP-XhoI-Rev. *CLIP^{Cd}* was PCR-amplified from pMS516 (38) using the primer pair CLIP-SOE-Fw/ CLIP-NotI-Rev. All the promoters were PCR-amplified from the genomic DNA of *C. difficile* R20291 using the following pair of primers: *cdtR*-EcoRI-Fw/ *cdtR*-SOE-Rev, *cdtA*-EcoRI-Fw/ *cdtA*-SOE-Rev, *tcdA*-SNAP-EcoRI-Fw/ *tcdA*-XhoI-Rev and *tcdA*-SNAP-EcoRI-Fw/ *tcdA*-CLIP-SOE-Rev. Using overlap extension PCR, the promoters were fused to the respective reporter tag to produce P_{cdtR}-*SNAP^{Cd}*: 1583 bp; P_{cdtA}-*SNAP^{Cd}*: 1067 bp; P_{tcdA}-*SNAP^{Cd}*: 1549 bp; P_{tcdA}-*CLIP^{Cd}*: 1551 bp. All the *SNAP^{Cd}* fusions were inserted between the EcoRI/ XhoI sites of pMTL84121 (60), while the *CLIP^{Cd}* fusion was inserted between EcoRI/ NotI sites of pMTL84121, to yield plasmids pSR9, pSR10, pSR17 and pSR12, respectively.

Mutant construction through allelic exchange in *C. difficile*. *tcdR* and *cdtR* in-frame deletion mutants were generated using allele-couple exchange (ACE) in *C. difficile* R20291Δ*pyrE* as described (54). Plasmids pSR1 and pSR11 were originated from pMTL-YN4 and used to construct *tcdR* and *cdtR* mutants, respectively. The homology regions upstream and downstream of the desired junction point within *tcdR* and *cdtR* were amplified by overlap extension PCR using the following primer pairs: tcdR-AscI-Fw/ tcdR-SOE-Rev and tcdR-SOE-Fw/ tcdR-SbfI-Rev; cdtR-AscI-Fw/ cdtR-SOE-Rev and cdtR-SOE-Fw/ cdtR-SbfI-Rev. The size of the final product that was cloned to originate pSR1 was 1192 bp, while the one that was inserted to originate pSR11 was 1382 bp. Both fragments were inserted between the SbfI and AscI sites of pMTL-YN4. pSR1 and pSR11 were

transformed into *E. coli* HB101 (RP4) and transferred to *C. difficile* R20291Δ*pyrE* by conjugation. Conjugants were selected by sub-culturing on BHI agar containing thiamphenicol. Resistant colonies were screened for single cross-over mutants by DNA extraction (5% chelex from Sigma-Aldrich) and PCR amplification of the regions surrounding the interested gene using the primer pairs tcdR-vef-Fw/ YN4-Fw and tcdR-vef-Rev/ YN4-Rev, cdtR-vef-Fw/ YN4-Fw and cdtR-vef-Rev/ YN4-Rev. The clones that successfully integrated the plasmid were streaked onto CDMM supplemented with 5-Fluoroorotic acid (2 mg/mL) and uracil (5 µg/mL) in order to select for plasmid excision. The resistant colonies were tested again by DNA extraction and PCR using the primer pairs: tcdR-vef-Fw/ tcdR-vef-Rev and cdtR-vef-Fw/ cdtR-vef-Rev. After plasmid loss confirmation, the *pyrE*$^-$ gene was reverted through conjugation of pMTL-YN2C. The clones that were able to grow onto non-supplemented CDMM were tested by DNA extraction and PCR using the pair of primers pyrE-Fw/ pyrE-Rev, yielding AHCD1102 (*tcdR* mutant) and AHCD1103 (*cdtR* mutant). Plasmid loss was confirmed through thiamphenicol susceptibility. The Δ*pyrE* gene from R20291Δ*pyrE* was also reverted in the same way as described for the mutants, originating strain AHCD1180.

Gene complementation through allelic exchange at the *pyrE* locus. To complement *tcdR* and *cdtR* mutations, the coding sequences and its promoter regions (1480 bp and 1727 bp, respectively) were amplified by PCR using primer pairs tcdR-comp-BamHI-Fw/ tcdR-comp-HindIII-Rev and cdtR-comp-BamHI-Fw/ cdtR-comp-XhoI-Rev. The fragments were then digested with BamHI and HindIII or XhoI and inserted between the same sites of pMTL-YN2C (54), yielding pSR34 and pSR35. These plasmids were introduced into *E. coli* HB101 (pRP4) and then transferred to strains R20291Δ*tcdR*Δ*pyrE* or R20291Δ*cdtR*Δ*pyrE* by conjugation. After selection for cells that were resistant to thiamphenicol, clones were streaked

onto non-supplemented CDMM. Colonies that were able to grow in the absence of uracil were tested by DNA extraction (5% chelex from Sigma-Aldrich) and PCR. Primers pyrE-Fw/ pyrE-Rev were used to confirm *pyrE* reversion and gene complementation at the *pyrE* locus. Clones that were positives for gene complementation were identified and named AHCD1161 (*pyrE::tcdR*) and AHCD1312 (*pyrE::cdtR*).

The $cdtR^{D61E}$ fragment was obtained by overlap extension PCR using the primer pairs cdtR-comp-BamHI-Fw/ cdtRD61E-SOE-Rev and cdtRD61E-SOE-Fw/ cdtR-comp-XhoI-Rev. The final PCR was performed with the primer pair cdtR-comp-BamHI-Fw/ cdtR-comp-XhoI-Rev producing a fragment with 1727 bp which was inserted between the BamHI and XhoI sites of pMTL-YN2C originating pSR46. This plasmid was transformed in *E. coli* HB101 (RP4) and subsequently transferred to *C. difficile* R20291*ΔcdtRΔpyrE* and R20291*ΔpyrE*. After selection for cells that were resistant to thiamphenicol, clones were streaked onto non-supplemented CDMM. Colonies that were able to grow in the absence of uracil were tested by DNA extraction (5% chelex from Sigma-Aldrich) and PCR. The pair of primers pyrE-Fw and pyrE-Rev was used to confirm *pyrE* reversion and gene complementation at the *pyrE* locus. Clones that were positive for gene complementation were named AHCD1313 (R20291Δ*cdtR pyrE*::$cdtR^{D61E}$) and AHCD1338 (R20291 *pyrE*::$cdtR^{D61E}$).

AlphaFold2 modelling. AlphaFold2 was used for the structural modelling of CdtR (61). The models were obtained by uploading the sequencs to the open access AlphaFold2 Colab notebook (https://colab.research.google.com/github/deepmind/alphafold/blob/main/notebooks/AlphaFold.ipynb; (62)). Five models were generated for each protein or complex. The confidence of the modelling was assessed by the pLDDT metric and the predicted

alignment error (PAE), *i.e.*, the uncertainty about the interface. Values of pLDDT > 90 are expected to be highly accurate. Only the best models are represented in Fig. S1. Structural representations were generated using PyMOL Molecular Graphics System (Schrödinger, LLC).

Supplemental Fig. Legends

Figure S1 - A: Alignment of *C. difficile* CdtR (from strain 630: CD630_24901, UNIPROT Q182U3; from strain R20291, CDR20291_2490/UNIPROT A0A9R0BLY1), the the CD630_26010 (UNIPROT Q182U2) and CDR20291_2488 (UNIPROT A0A9R0CF96) proteins (see Fig. 1) with selected orthologue: LytT from *B. subtilis*, LytR (Q2FK09), AgrA (P0A0I7-1) from *S. aureus* and *S. pneumoniae* ComE (B2INQ3). The alignment was generated using Clustal Ω (https://www.ebi.ac.uk/jdispatcher/msa/clustalo) and represented using Jalview (https://www.jalview.org). The following alignment parameters are shown: quality (a measure of the likelihood of finding mutations at a particular position), conservancy (which reflects the conservation of the chemical properties of the residues for each column), the consensus and a sequence logo. In ComE, the aspartate residue at position 58 (D58; black dot) is phosphorylated by the ComD kinase whereas the threonine residue 128 (T128; green dot) is phosphorylated by the StkP kinase (41, 52). The residue homologous to D58 in the two CdtR proteins is D61 (red dot); T128 is conserved in AgrA but not in the other proteins included in the alignment. The limits of the receiver (REC) and LytR-like DNA-binding domains are represented as well as the linker between the two domains. The residues marked by the blue dots are involved in DNA interactions as shown in the crystal structure of the LytR domain of AgrA in complex with a DNA binding site (see also **D**). **B:** shows the crystal structure of a *S. pneumoniae* ComE dimer (pdb code: 4cbv) (34). **C**: shows an AlphaFold2 model of a CdtR dimer (from strain R20291). In **B** and **C**, the REC and LytR domains are indicated as well as the N- and C-termini of the two proteins; the region of the REC domain with the phosphor-acceptor aspartate is encircled and the residue highlighted in yellow. Residues predicted to be involved

in DNA binding (see also panel **D**) are indicated. The grey arrows indicate the orientation of the two LytR-like domains, compatible with binding of two direct repeats on the DNA. Also note the reverse orientation of the LytR domain of CdtR, relative to the REC domain, in comparison with ComE. The broken line represents the symmetry axis. **D**: structure of one of the LytR domains of the *S. aureus* AgrA protein (pdb code: 3bs1). The residues highlighted are involved in direct base-specific contacts bases in consecutive major grooves (H169 and R233) and the intervening minor groove (N201) (47, 63); only H169 and R233, which are located in the loops connecting the ß strands that form the LytR fold, are essential for DNA binding (47).

Figure S2 . Growth curves and sampling. Cultures of the strains bearing the indicated transcriptional fusion to the SNAP^{Cd} reporter were grown in TY medium and samples collected at the indicated time points (arrowheads). Representative growth curves are shown. See also Fig. 2 and the main text for details.

Figure S3 . Background levels of fluorescence in a Pl$_{ess}$-*SNAP*^{Cd} strain. A: Samples were withdrawn at the indicated times from TY cultures of R20291 derivatives carrying a plasmid with a promoter-less *SNAP*^{Cd} reporter. Cells were labelled with the SNAP-tag substrate and imaged by fluorescence microscopy. Fluorescence intensity (in arbitrary units, AU) was quantified from microscopy images. The numbers indicate the fluorescence intensity values below which 99% of the data fall. At least 100 cells were analyzed. Data shown are from a representative experiment. **B:** Microscopy analysis of *C. difficile* cells producing SNAP under the control of P$_{cdtA}$-*SNAP*^{Cd} (blue arrowheads). Cells were labelled with TMR-Star and mixed with cells of a strain carrying a promoter-less *SNAP*^{Cd} reporter (yellow arrowheads), labelled in the same conditions but subsequently stained with DAPI to allow distinction between the two cell types.

A higher background fluorescence is observed in cells labeled with TMR-Star at 16–24 hours compared to 8 hours. However, in contrast to the homogeneous signal observed in cells expressing SNAP (blue arrow), cells that do not express SNAP show non-specific binding of TMR-Star to the bacterial cell, resulting in a reticular signal (yellow arrow; cells stained with DAPI). Scale bar: 1 μm. **C:** Whole-cell extracts were prepared from the strains imaged in panel B immediately after labeling with TMR-Star, indicated by "+TMR" ("−TMR" indicates unlabeled samples). Five micrograms of total protein were resolved by SDS-PAGE. Gels were scanned using a fluorimager (middle panel), stained with Coomassie (upper panel), or subjected to immunoblotting with anti-SNAP antibodies (bottom panel). Black and red arrows indicate unlabeled and TMR-Star-labeled SNAP, respectively. The positions of molecular weight markers (in kDa) are indicated. In strains lacking SNAP expression, no covalent labeling of other proteins is detected, indicating that the background fluorescence in panel B results from non-specific TMR-Star binding.

Figure S4 - *cdtR* and *tcdR* mutants. Allele Coupled Exchange mutagenesis (ACE) to produce *cdtR* (**A**) and *tcdR* (**B**) mutants in *C. difficile* strain R20291. The CdtLoc (**A**) and *tcdR*/*tcdB* (**B**) region are represented together with the position of the sequence deleted. PCR amplification using the cdtR-vef-Fw (P1 in the Fig.) and cdtR-vef-Rev (P2) primers allows identification of a WT (1815 bp) or an in-frame deleted *cdtR* (1414 bp), while amplification using primers tcdR-vef-Fw (P3) and tcdR-vef-Rev (P4) allows identification of the WT (2371 bp) or the in-frame deleted *tcdR* (1915 bp). The agarose gels below each panel shows the electrophoretic analysis of the PCR products amplified from the WT and deletion mutants.

Supplemental Tables

Table S1 - Bacterial strains used in this study.

Strain	Relevant properties	Origin
E. coli		
DH5α	General cloning host	Invitrogen
HB101 (RP4)	Host for conjugation into *C. difficile*	Laboratory stock
AHEC095	HB101 (RP4)/ pFT46	(36)
AHEC165	HB101 (RP4)/ pFT73	(36)
AHEC227	HB101 (RP4)/ pSR1	This study
AHEC450	HB101 (RP4)/ pSR10	"
AHEC470	HB101 (RP4)/ pSR9	"
AHEC490	HB101 (RP4)/ pSR12	"
AHEC495	HB101 (RP4)/ pSR11	"
AHEC508	HB101 (RP4)/ pSR17	"
AHEC661	HB101 (RP4)/ pSR34	"
AHEC686	HB101 (RP4)/ pSR35	"
AHEC694	HB101 (RP4)/ pSR46	"
AHEC698	HB101 (RP4)/ pSR37	"
C. difficile		
AHCD586	630Δ*erm* carrying pFT46	(36)
AHCD646	R20291 pFT47	"
AHCD714	630Δ*erm* carrying pFT73	"
AHCD774	R20291Δ*pyrE*	(54)
AHCD1102	R20291Δ*tcdR*	This study
AHCD1103	R20291Δ*cdtR*	"
AHCD1116	R20291Δ*tcdR* containing pSR9	"
AHCD1117	R20291Δ*tcdR* containing pSR10	"
AHCD1119	R20291Δ*cdtR* containing pSR10	"
AHCD1124	R20291Δ*cdtR* containing pSR9	"
AHCD1161	R20291Δ*tcdR pyrE*::*tcdR*	"
AHCD1164	R20291Δ*tcdR pyrE*::*tcdR* containing pSR10	"
AHCD1180	R20291 *pyrE*$^{+}$	"
AHCD1184	R20291 *pyrE*$^{+}$ containing pSR9	"
AHCD1189	R20291 *pyrE*$^{+}$ containing pSR10	"
AHCD1211	R20291 *pyrE*$^{+}$ containing pSR17	"
AHCD1260	R20291Δ*cdtR* containing pSR17	"
AHCD1312	R20291Δ*cdtR pyrE*::*cdtR*	"
AHCD1313	R20291Δ*cdtR pyrE*::*cdtR*^{D61E}	"
AHCD1315	R20291Δ*cdtR pyrE*::*cdtR* containing pSR10	"
AHCD1316	R20291Δ*cdtR pyrE*::*cdtR* containing pSR17	"
AHCD1319	R20291Δ*cdtR pyrE*::*cdtR*^{D61E} containing pSR10	"
AHCD1321	R20291Δ*cdtR pyrE*::*cdtR*^{D61E} containing pSR17	"
AHCD1338	R20291 *pyrE*::*cdtR*^{D61E}	"
AHCD1360	R20291 *pyrE*::*cdtR*^{D61E} containing pSR10	"
AHCD1362	R20291 *pyrE*$^{+}$ containing pSR12	"

Table S2 - Oligonucleotides used in this study.

Primer	Sequence (5' to 3')
cdtR-EcoRI-Fw	CCCGAATTCCTAATGCTATACAGACCAAGTC
cdtR-SOE-Rev	ATTTCACAATCTTTATCCATTAAATACCCTCC
SNAP-SOE-Fw	ATGGATAAAGATTGTGAAATGAAGAGAACC
SNAP-XhoI-Rev	CCGCTCGAGTTACCCAAGTCCTGGTTTCCCCAAACG
cdtAB-EcoRI-Fw	CCCGAATTCCATATTGTTTCTCTCGACC
cdtAB-SOE-Rev	ATTTCACAATCTTTATCCATTTATTCTCCCTCCC
tcdA-SNAP-EcoRI-Fw	CACAAAGATGAATTCTGGTCAGTTGGT
tcdA-XhoI-Rev	CGCCTCGAGTTACTGTAATGCTTCAGTGG
tcdA-CLIP-SOE-Rev	CGCAGTCTTTATCCATTTACTGTAATGCTTCAGTGG
CLIP-SOE-Fw	ATGGATAAAGACTGCGAGATGAAACG
CLIP-NotI-Rev	CCCGCGGCCGCTTATCCTAATCC
tcdR-AscI-Fw	CCCCGGCGCGCCATTATCTTAAGAGAGGAG
tcdR-SOE-Rev	CATAAATAAAATTTCTTGCAAATCATC
tcdR-SOE-Fw	TTGCAAGAAATTTTATTTATGGAAAATTATTTTAACTTG
tcdR-SbfI-Rev	CCCCCCTGCAGGTATCTATATAAATATCTG
tcdR-comp-BamHI-Fw	CCCGGATCCTAAAAATATTTTGATATG
tcdR-comp-HindIII-Rev	CCCAAGCTTATTAATTTGCTCTTC
tcdR-vef-Fw	GTATCATTTCACGAAGAGG
tcdR-vef-Rev	GGGTCATTTAAGTTTTCTC
cdtR-AscI-Fw	CCCCGGCGCGCCGTGCTAAACACAC
cdtR-SOE-Rev	TTGTGCTATCCATAATCCATCACATAATTC
cdtR-SOE-Fw	GGATTATGGATAGCACAACATATTGTTTCTCTC
cdtR-SbfI-Rev	CCCCCCTGCAGGGTAAGTCTTGTGCATAAATG
cdtRD61E-SOE-Rev	CTTGTATTTTGCCTCTACAAC
cdtRD61E-SOE-Fw	GTAGAGGCAAAATACAAGATAAG
cdtR-vef-Fw	CAATGTTATGTAGATTCTTAC
cdtR-vef-Rev	GGAGCCTTGTAAGTAGTATTGC
cdtR-comp-BamHI-Fw	CCCGGATCCGAAGTAATCCTATCTCGAC
cdtR-comp-XhoI-Rev	CCCCTCGAGCAATGGTTGCAATGTCTAATAG
YN4-Fw	CAAGAAGAGCGACTTCGCGGAGCTGG
YN4-Rev	CCATTACAGACTTATCCAGGG
PyrE-Fw	GAGGAATTATATAAGGTAGAATAAATAG
PyrE-Rev	CTAACCTTCATTTATAAGGCTAACTGC

[a] engineered restriction sites are underlined.

Table S3 - Plasmids used in this study.

Plasmid	Relevant properties	Origin
pMTL84121	*Clostridium* modular plasmid containing *catP* (Cm^R/ Tm^R)	Laboratory stock
pFT46	*Ptet-SNAP^{Cd}* in pMTL84121	(36)
pFT47	SNAP^{Cd} in pMTL84121	(36)
pFT73	*Ptet-CLIP^{Cd}* in pMTL84121	“
pMS516	*CLIP^{Cd}* in pMTL84121	(38)
pMTL-YN4	Cloning vector for Allele Coupled Exchange in *C. difficile* (R20291) containing the *pyrE* allele (Cm^R/Tm^R)	(54)
pMTL-YN2C	Cloning vector for gene complementation and *pyrE* reversion through Allele Coupled Exchange (Cm^R/Tm^R)	“
pSR1	Δ*tcdR* pMTL-YN4	This study
pSR9	P*cdtR*-SNAP^{Cd} in pMTL84121	"
pSR10	P*cdtAB*-SNAP^{Cd} in pMTL84121	"
pSR11	Δ*cdtR* pMTL-YN4	"
pSR12	P*cdtAB*-SNAP^{Cd} and P*tcdA*-CLIP^{Cd} in pMTL84121	"
pSR17	P*tcdA*-SNAP^{Cd} in pMTL84121	"
pSR34	pMTL-YN2C containing *tcdR* and its promoter for complementation	"
pSR35	pMTL-YN2C containing *cdtR* and its promoter for complementation	"
pSR46	pMTL-YN2C containing *cdtR*^{D61E} and its promoter for complementation	"

Ramalhete *et al.*, FIG. S1

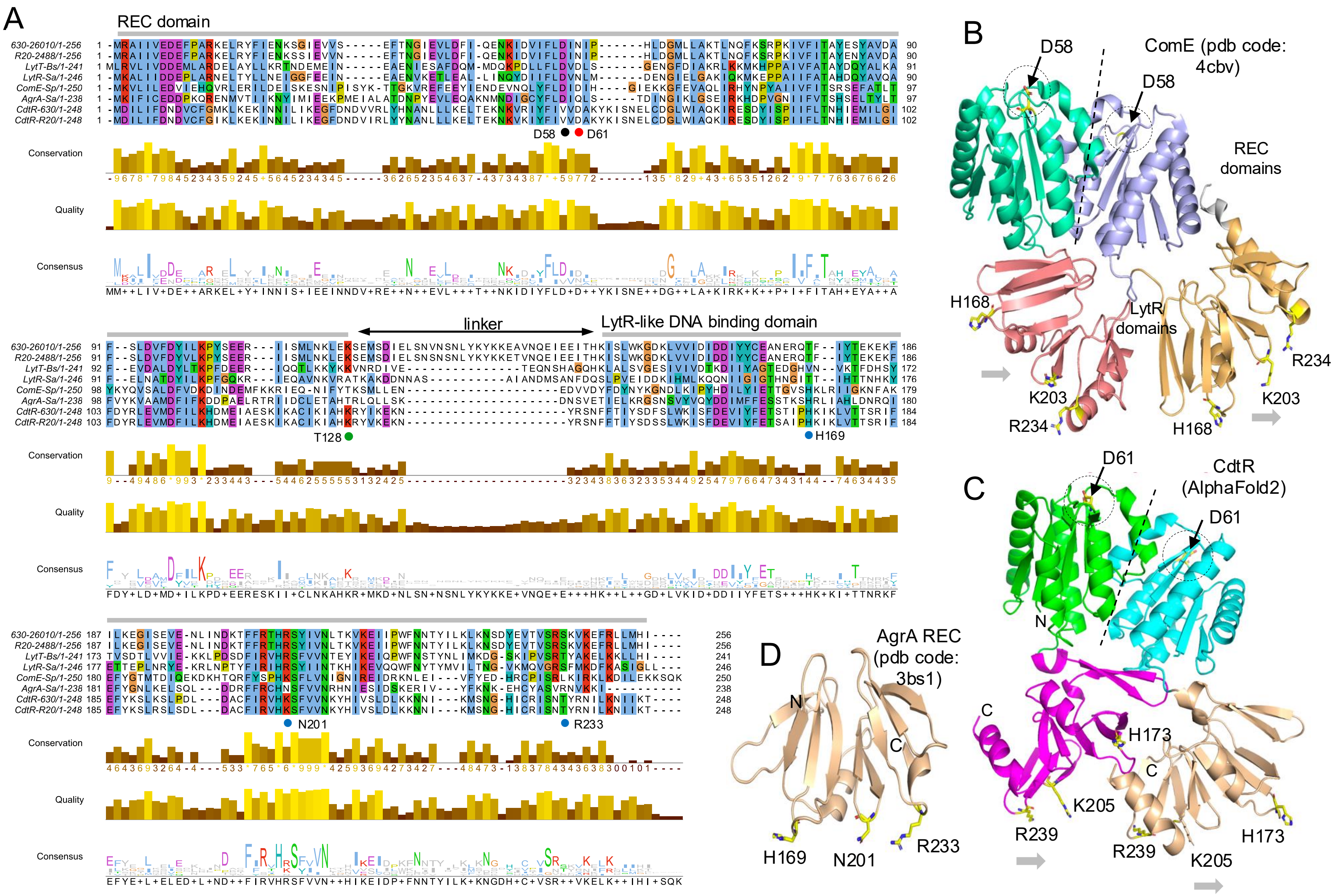

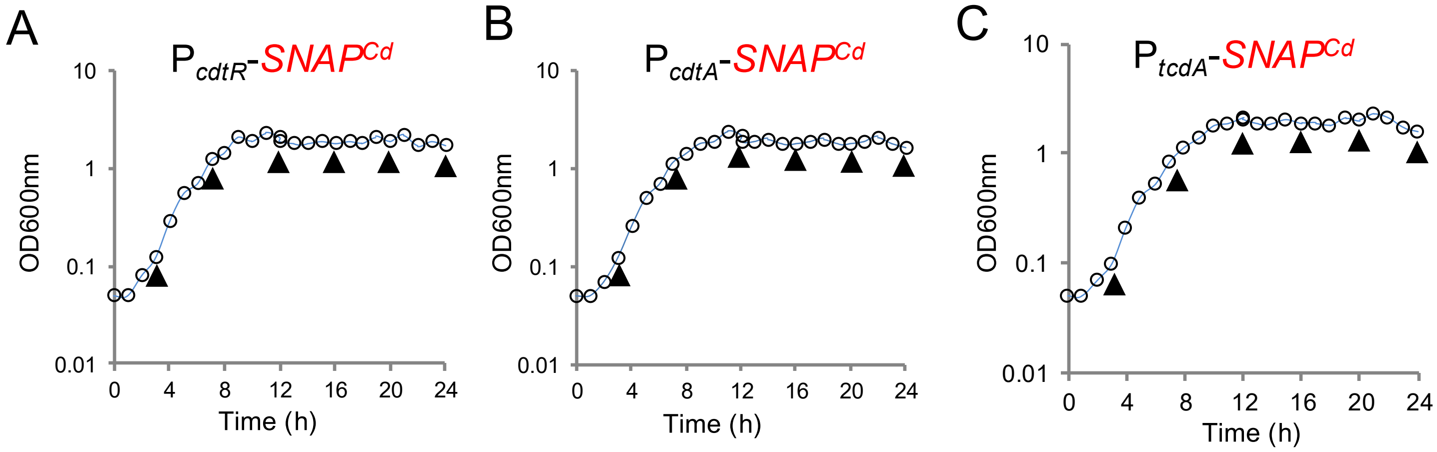
A
P_{cdtR}-$SNAP^{Cd}$
OD600nm
10
1
0.1
0.01
0
4
8
12
16
20
24
Time (h)
B
P_{cdtA}-$SNAP^{Cd}$
OD600nm
10
1
0.1
0.01
0
4
8
12
16
20
24
Time (h)
C
P_{tcdA}-$SNAP^{Cd}$
OD600nm
10
1
0.1
0.01
0
4
8
12
16
20
24
Time (h)

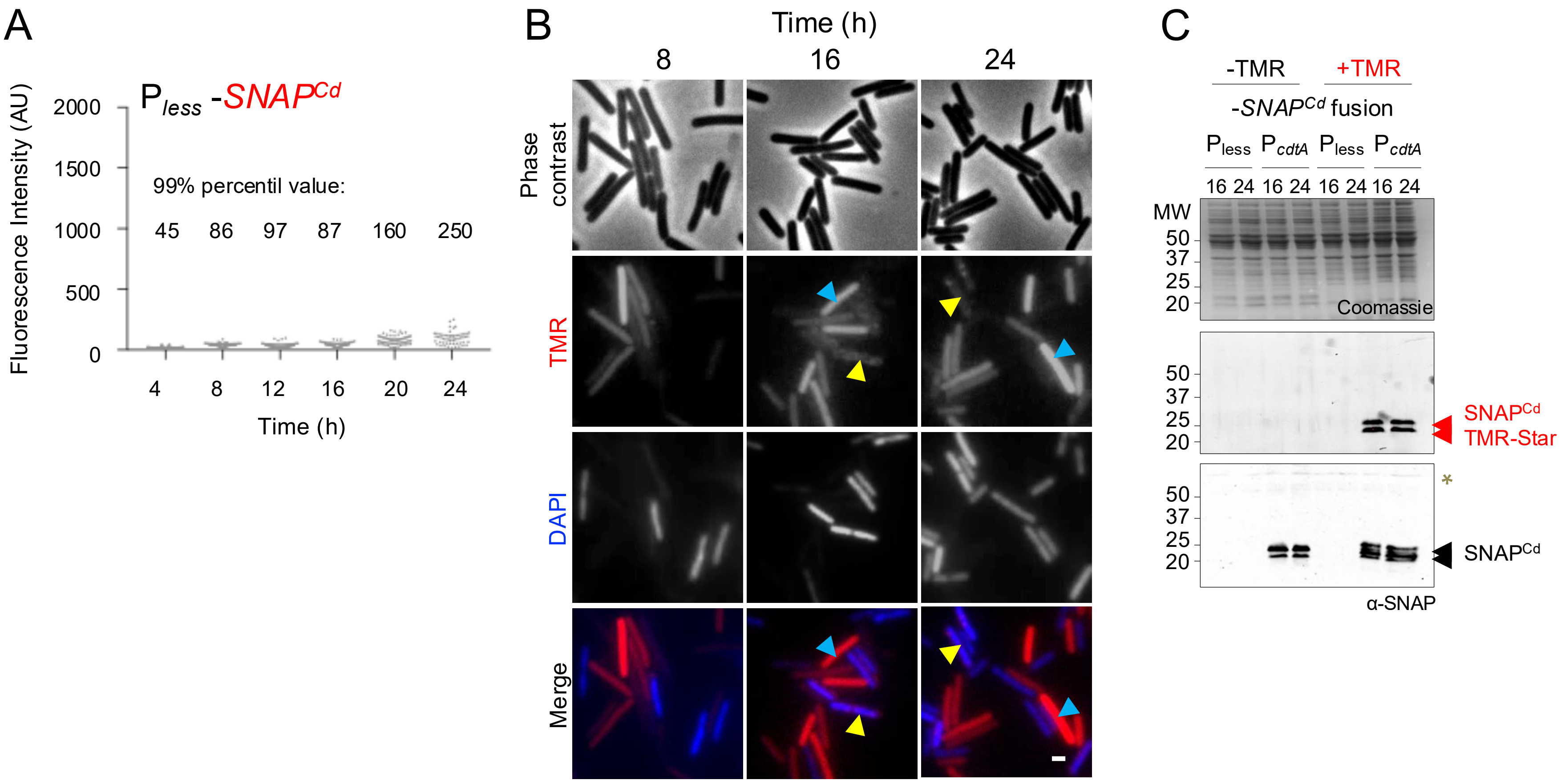
A
B
C
P_{less} -$SNAP^{Cd}$
99% percentil value:
45 86 97 87 160 250
Fluorescence Intensity (AU)
2000
1500
1000
500
0
4 8 12 16 20 24
Time (h)
Time (h)
8 16 24
Phase contrast
TMR
DAPI
Merge
-TMR +TMR
-$SNAP^{Cd}$ fusion
P_{less} P_{cdtA} P_{less} P_{cdtA}
16 24 16 24 16 24 16 24
MW
50
37
25
20
Coomassie
$SNAP^{Cd}$ TMR-Star
*
$SNAP^{Cd}$
α-SNAP

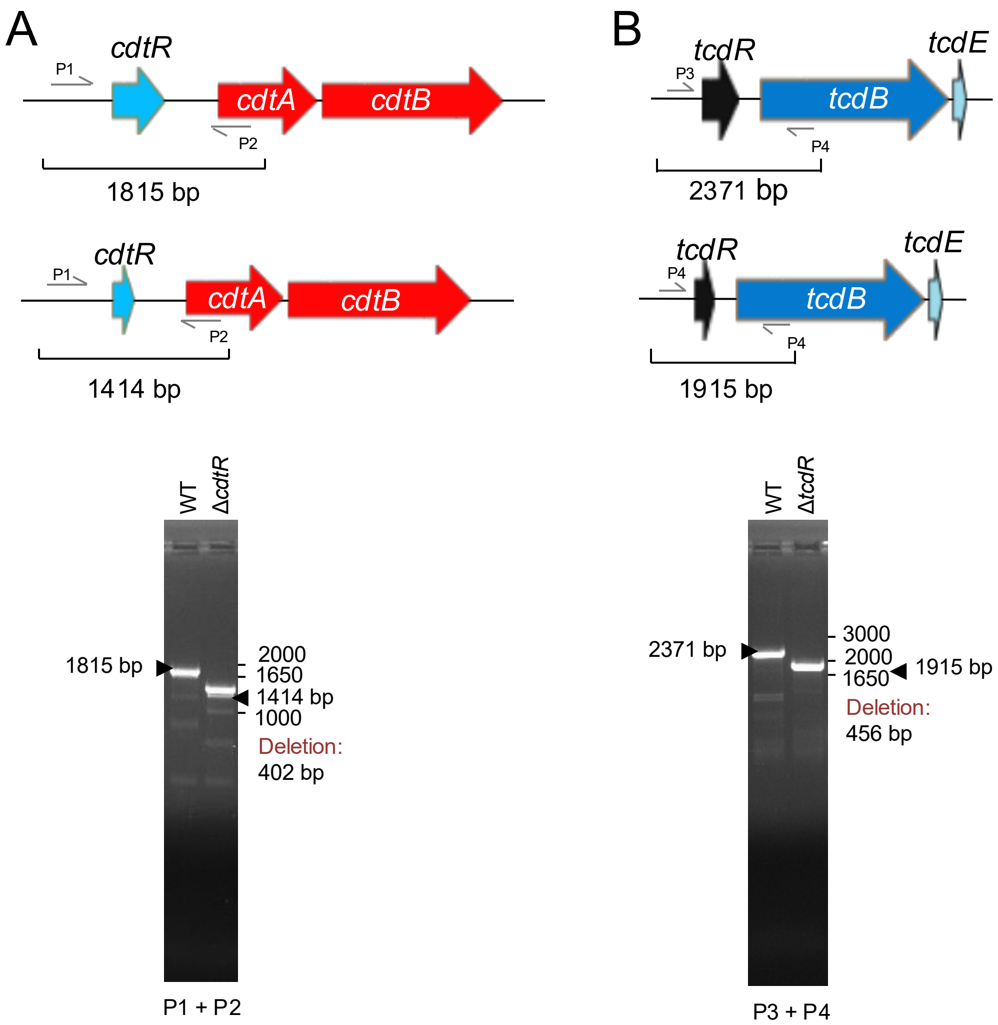
A
cdtR
P1
cdtA
cdtB
P2
1815 bp
cdtR
P1
cdtA
cdtB
P2
1414 bp
WT
ΔcdtR
1815 bp
2000
1650
1414 bp
1000
Deletion:
402 bp
P1 + P2
B
tcdR
tcdE
P3
tcdB
P4
2371 bp
tcdR
tcdE
P4
tcdB
P4
1915 bp
WT
ΔtcdR
3000
2371 bp
2000
1650
1915 bp
Deletion:
456 bp
P3 + P4
